# Clinical feature and genetic analysis of *HMBS* gene in Chinese patients with acute intermittent porphyria: a systematic review

**DOI:** 10.3389/fgene.2023.1291719

**Published:** 2023-12-11

**Authors:** Yi Ren, Shuang Li, Jia-Jia Lei, Ru Li, Bai-Xue Dong, Jing Yang

**Affiliations:** ^1^ Department of Endocrinology, The First Hospital of Shanxi Medical University, Taiyuan, China; ^2^ Department of First Clinical Medical School, Shanxi Medical University, Taiyuan, China

**Keywords:** acute intermittent porphyria, hydroxymethylbilane synthase, China, mutation analysis, abdominal pain

## Abstract

**Background:** Early detection and diagnosis are important crucial to prevent life-threatening acute attacks in patients with acute intermittent porphyria (AIP). We aim to provide comprehensive data on the clinical and *hydroxymethylbilane synthase (HMBS)* gene variant characteristics and genotype-phenotype association of Chinese patients with AIP in order to improve clinicians’ knowledge of AIP and reduce misdiagnosis and mistaken treatment.

**Methods:** We searched the literature on Chinese patients with AIP in PubMed, Web of Science, Wiley Online Library, ScienceDirect and Chinese literature databases up to August 2023 in our analysis to explore the clinical and *HMBS* gene variant characteristics of Chinese patients with AIP.

**Results:** A total of 41 original articles associated with Chinese AIP patients were included for analysis: 97 variants were detected in 160 unrelated families, including 35 missense, 29 frameshift, 24 splicing and 9 nonsense variants, with c.517C>T being the most common variant. Clinical data were reported in 77 of 160 patients: Most of them were female (67/77) and the age was 28.8 ± 9.9 years. The most common symptom was abdominal pain (73/77, 94.8%), followed by central nervous system symptoms (45/77, 58.4%). 13.0% (10/77) of patients experienced psychiatric symptoms. Hyponatremia was the most common electrolyte abnormality (42/77). 31 patients received carbohydrate loading therapy, and 30 of them were improved. 6 patients were treated with carbohydrate loading combined with hemin therapy and 5 eventually improved. All variants causing premature stop codons, frameshifts or enzyme activity center may experience more severe clinical phenotypes such as seizures, respiratory paralysis, intracranial hemorrhage disorder or respiratory failure.

**Conclusion:** The most common presenting symptom in Chinese AIP patients was abdominal pain, followed by central nervous system symptoms. The *HMBS* gene analysis in Chinese AIP patients revealed that the heterogeneity is strong and the most common variant was missense mutation, with c.517C>T being the most common variant. The genotype-phenotype association helps guide clinical diagnosis and treatment. However, the treatment for AIP in China is limited and monolithic, and more attention needs to be paid to the treatment.

## 1 Introduction

Acute intermittent porphyria (AIP) is an autosomal dominant disorder caused by partial deficiency of the third enzyme, hydroxymethylbilane synthase (HMBS), in heme synthesis (Stölzel et al., 2021). It has a low penetrance of only 1% based on all AIP heterozygotes (Chen et al., 2016). Most carriers remain disease-free for life and are known as latent AIP, while some patients experience life-threatening acute attacks, known as manifest AIP, due to common factors such as menstruation, smoking, drinking, infection, fasting and drug. The clinical manifestations of acute attacks of AIP are complex and varied involving multiple systems such as the gastrointestinal, neurological, and psychiatric systems. And there is substantial heterogeneity in severity, even in the same family.

The *HMBS* gene is considered to be the only gene responsible for the disease. Its housekeeping transcript consists of 14 exons and corresponding introns. Its pathogenic variant can lead to functional defects of HMBS. Genetic screening provides 95% sensitivity and about 100% specificity, which has been rapidly incorporated into good clinical practice (Kauppinen, 2004). It not only can diagnose the manifest AIP, but is also one of the most accurate methods to screen latent AIP. It has great significance for the early diagnosis of high-risk groups, effective prevention of acute attacks and improvement of the patients’ lives. With the development of gene sequencing test, numerous of mutations and their linked phenotypes have been identified, which has helped to establish genotype-phenotype correlations.

At present, there is a lack of epidemiological data on AIP worldwide, but Europe performed large scale prospective study to investigate the incidence of porphyrias (Elder et al., 2013). And multiple countries such as South Africa (Fortgens et al., 2017), the United States (Bonkovsky et al., 2014), Argentina (Cerbino et al., 2015), Colombia (Jaramillo-Calle and Aguirre Acevedo, 2019), and Russia (Goncharova et al., 2019) have reported cohort studies on AIP. And with the development of sequencing technology, more and more AIP patients have been reported in China. However, most of them are case reports, and there is still a lack of systematic analysis of the characteristics of Chinese AIP patients. A previous study identified 5 pathogenic and 20 likely pathogenic variants from the ChinaMAP database and preliminarily analyzed the epidemiological features of AIP in Hebei Province, China (Ma et al., 2022). However, there is insufficient knowledge and research on AIP in China, which can easily lead to misdiagnosis and mistaken treatment in clinical practice. How to provide timely and accurate diagnosis and treatment is a major challenge for healthcare worker. The aim of this study was to describe the clinical features and the characteristics of *HMBS* gene variants and genotype-phenotype association of Chinese AIP patients, in order to improve clinicians’ knowledge of AIP and to help clinicians in early identifying patients with this disease guide clinical management, and to provide genetic counselling and health education for asymptomatic heterozygotes.

## 2 Methods

### 2.1 Study design

This systematic review was conducted in accordance with the Preferred Reporting Items for Systematic Reviews and Meta-Analyses (PRISMA) statement checklist (Page et al., 2021) (Supplementary Material S1).

### 2.2 Strategy, criteria, and procedures for the literature search

We searched all literature about Chinese AIP patients for analysis in PubMed, Web of science, Wiley Online Library, ScienceDirect and Chinese databases CNKI, Wanfang and CQVIP up to August 2023. In the PubMed, Web of science, Wiley Online Library, ScienceDirect databases, we used the keywords “Acute Intermittent Porphyria” and “China or Chinese.” In the Chinese database, we used the keyword “Acute Intermittent Porphyria.” Inclusion criteria: (1) the cases were Chinese patients; (2) the diagnosis of AIP was confirmed by clinical and sequencing results (Zhang et al., 2020); (3) the variant was clearly reported. Exclusion criteria: (1) duplicated variant sites found in the same family or uncertain variant sites; (2) missing or uncomplete clinical data. The type of literature is not limited, all literature reporting correctly and complete information on AIP cases was accepted, such as original research, case reports, and briefs etc.

### 2.3 Data extraction

Data on the general information (age, sex, etc.), *HMBS* gene variants, clinical presentations, laboratory tests, treatment, and outcomes of the patients were retrieved. Microsoft Excel Spreadsheet software was used to organized and collated the extracted data. Two authors (SL, JL) identified the relevant original articles and extracted the data independently, while the third author (BD) checked the results. In case of disagreement, the relevant programs were repeated until a consensus was reached among the authors.

### 2.4 Data synthesis

The extracted data was then analyzed and interpreted by the SL and YR researchers. The primary outcomes assessed were the general information (hospital types, regions, age, sex, etc.), *HMBS* gene variants, clinical presentations, laboratory tests, treatment, and outcomes. A narrative (descriptive) method was conducted to synthesize this information.

### 2.5 The pathogenicity rating of *HMBS* variants and clinical phenotype

According to the standards and guidelines of American College of Medical Genetics and Genomics (ACMG) (Richards et al., 2015), each pathogenic criterion is weighted as very strong (PVS1), strong (PS1–4), moderate (PM1–6), or supporting (PP1–5). Each variant was classified for pathogenic (P), likely pathogenic (LP) and uncertain significance (VUS). AIP can present with a sudden life-threatening crisis characterized by severe abdominal pain and neuropsychiatric symptoms (Bissell et al., 2017). According to the main manifestation, the severity of the disease was classified into mild (abdominal pain is the main clinical manifestation), moderate (accompanied by neuropsychiatric symptoms) and severe (experiencing respiratory paralysis, intracranial hemorrhage, disseminated intravascular coagulation (DIC), acute heart failure (AHF), chronic renal failure (CRF) or respiratory failure, etc).

## 3 Results

### 3.1 Search results

**FIGURE 1:**
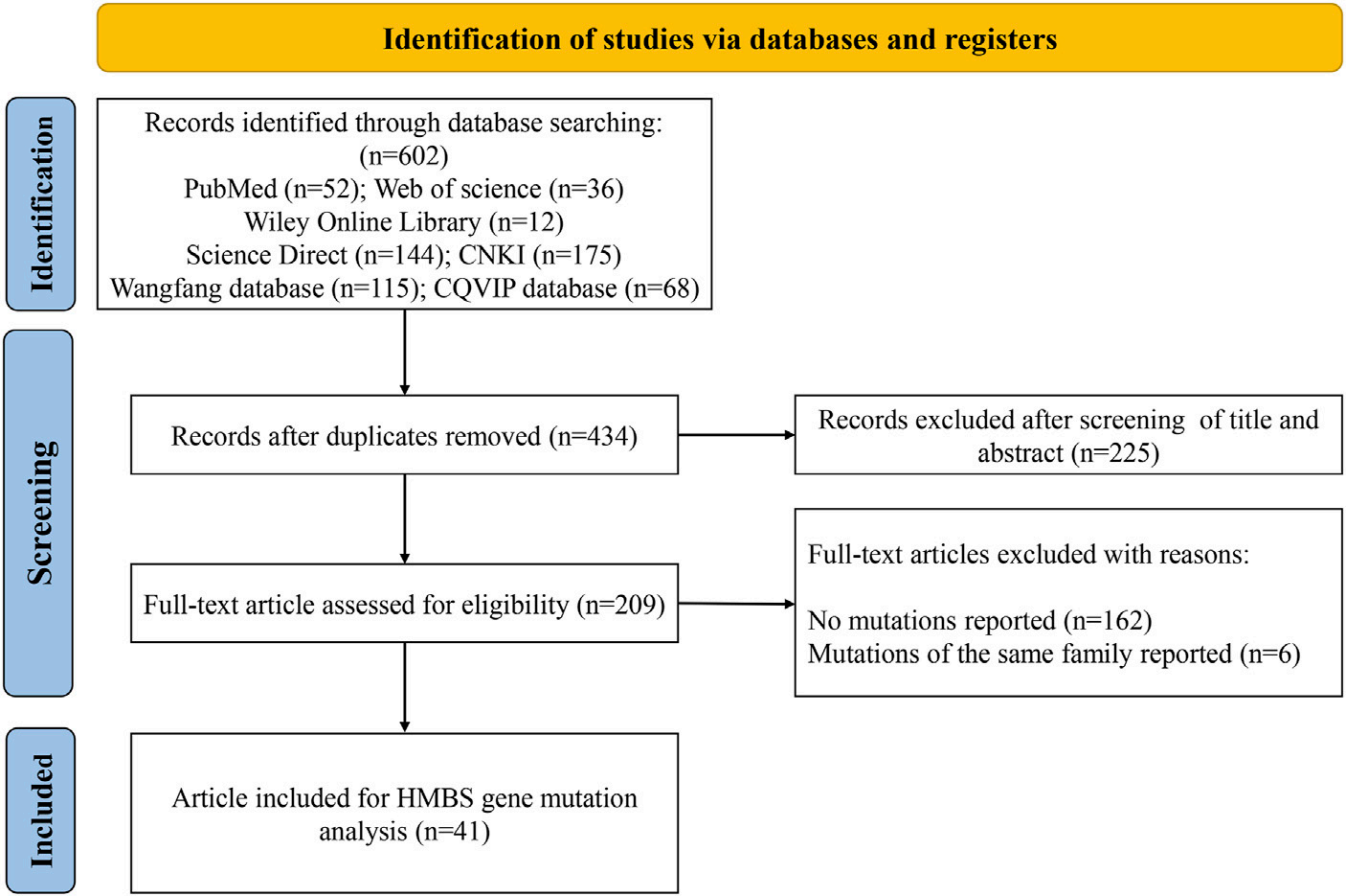
Flow chart of the study selection process.

The screening process is shown in Figure 1. A total of 602 publications were identified after searching PubMed (*n* = 52), Web of Science (*n* = 36), Wiley Online Library (*n* = 12), ScienceDirect (*n* = 144) and Chinese databases CNKI (*n* = 175), Wanfang (*n* = 115) and CQVIP (*n* = 68). After removing duplicates, titles and abstracts of 434 publications were screened. The full text of the remaining 209 studies was downloaded and evaluated, and another 168 studies were further excluded due to no variants reported or variants of the same family reported. Finally, 41 studies (Lam et al., 2001; Yang et al., 2008; Lam et al., 2011; Xie, 2012; Kong et al., 2013; Zhou, 2014; Li et al., 2015; Li et al., 2015; Chen et al., 2015; Jiao et al., 2015; Yang et al., 2015; You et al., 2015; Yuan et al., 2015; Li et al., 2016; Yang et al., 2016; Lei et al., 2017; Li et al., 2017; Yang et al., 2017; Hu et al., 2018a; Zheng et al., 2018; Wang et al., 2019; Wang et al., 2019; Hu, 2019; Zhang and Gao, 2019; Yang et al., 2020; Yang et al., 2020; Zhang et al., 2020; Sun et al., 2020; Teng et al., 2020; Fu et al., 2021; Gao et al., 2021; Huang et al., 2021; Zhang et al., 2021; Haiqing, 2022; Hu et al., 2022; Li, 2022; Li et al., 2022; Yang et al., 2022; Zhou et al., 2022; Guo and Luo, 2023; Liang and Li, 2023) were obtained for analysis (21 English-language and 20 Chinese-language articles).

### 3.2 Genetic analysis of the HMBS gene in Chinese with AIP

**FIGURE 2:**
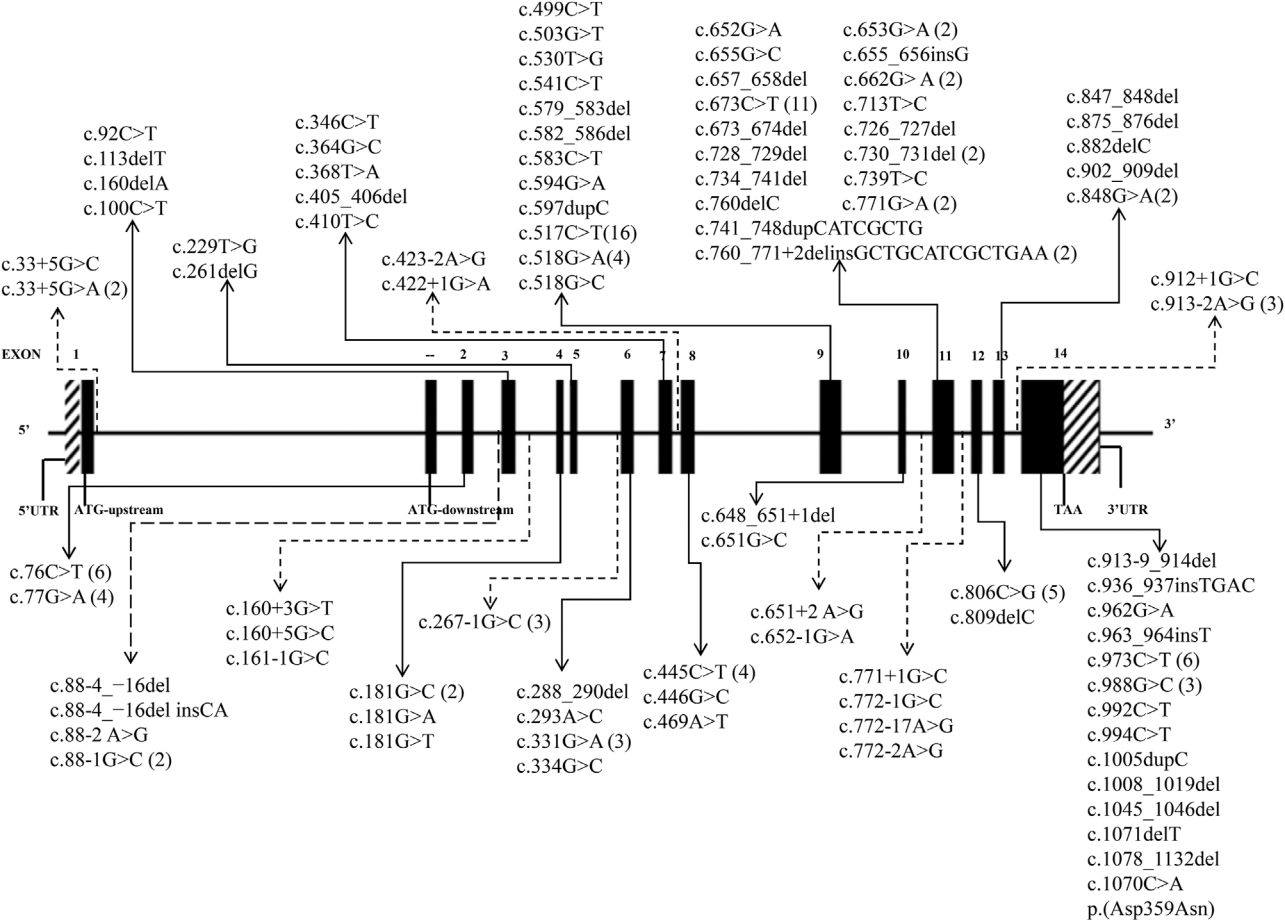
97 *HMBS* gene mutations spectrum in transcript (NM_000190.4). (The solid lines indicate exon mutation sites. Dashed lines indicate intron mutation sites. The parentheses indicate the frequency of mutations).

A total of 41 articles involving 160 patients were enrolled in the study, most of which were case reports. The majority of reports were sourced from grade III hospitals, predominantly originating from Hebei, Taiwan, and Beijing, with sporadic cases identified in other provinces. Totally, 97 variants were detected in 160 patients (Table 1), including 35 missense mutations (36.1%), 29 frameshift mutations (29.9%), 24 splicing mutations (24.7%) and 9 nonsense mutations (9.3%). Exon variants were mainly concentrated in exons 11 and 14 (34.0%). No variants were found in exon 1 (Figure 2). The majority of the mutations were family specificity, but 21.6% (21/97) variants occurred in several families, among these, c.517C>T was the most common variant, which was found in 16 unrelated families (Figure 2). According to the ACMG, among 97 variants, 45 variants are pathogenic, 14 variants are likely pathogenic and 38 variants are uncertain significance (Table 1).

**TABLE 1:** 97 *HMBS* gene mutations identified in 160 Chinese patients with AIP.

No.	Variants	References	Frequency	Position	Type	Pathogenicity	Evidences based on ACMG
1	c.33 + 5 G>C	Hu et al. (2022)	1	IVS 1	SD	VUS	PS3, PP4
2	c.33 + 5 G>A	Yang et al. (2008)	2	IVS 1	SD	VUS	PS3, PP4
3	c.76 C>T	Yang et al., 2008; Hu (2019), Liang and Li (2023)	6	Exon 2	MS	LP	PS3, PM1, PP3, PP4
4	c.77 G>A	Yang et al., 2008; Hu (2019)	4	Exon 2	MS	LP	PS3, PM1, PP3, PP4
5	c.88-16_88-4del	Yang et al. (2008)	1	IVS 2	SD	P	PVS1, PS3, PP4
6	c.88-16_88-4delinsCA	Yang et al. (2008)	1	IVS 2	SD	P	PVS1, PS3, PP4
7	c.88-2 A>G	Jiao et al. (2015)	1	IVS 2	SD	P	PVS1, PM2, PP4
8	c.88-1 G>C	Hu, 2019; Zhang et al. (2021)	2	IVS 2	SD	P	PVS1, PM2, PP4
9	c.92 C>T	Hu (2019)	1	Exon 3	MS	VUS	PP3, PP4
10	c.100 C>T	Lei et al. (2017)	1	Exon 3	NS	P	PVS1, PP3, PP4
11	c.113del	Yang et al. (2008)	1	Exon 3	FS	P	PVS1, PS3, PP4
12	c.160del	Yang et al. (2008)	1	Exon 3	FS	P	PVS1, PS3, PP4
13	c.160 + 3 G>T	Hu (2019)	1	IVS 3	SD	VUS	PM2, PP4
14	c.160 + 5 G>C	Li (2022)	1	IVS 3	SD	VUS	PS3, PM2, PP3, PP4
15	c.161-1 G>C	Li et al. (2015a)	1	IVS 3	SD	P	PVS1, PM2, PP4
16	c.181 G>C	Fu et al. (2021), Hu et al. (2022)	2	Exon 4	MS	VUS	PM5, PP3, PP4
17	c.181 G>A	Hu (2019)	1	Exon 4	MS	VUS	PM5, PP3, PP4
18	c.181 G>T	Hu (2019)	1	Exon 4	MS	LP	PS3, PM5, PP3, PP4
19	c.229 T>G	Hu (2019)	1	Exon 5	MS	VUS	PM2, PP3, PP4
20	c.261del	Hu et al. (2022)	1	Exon 5	FS	P	PVS1, PM6, PP4
21	c.267-1 G>C	Hu (2019)	3	IVS 5	SD	VUS	PVS1, PP4
22	c.288_290del	Guo and Luo (2023)	1	Exon 6	FS	P	PVS1, PM2, PP4
23	c.293 A>C	Hu (2019)	1	Exon 6	MS	VUS	PM2, PP3, PP4
24	c.331 G>A	Hu, 2019; Hu et al. (2022)	3	Exon 6	MS	LP	PS3, PP3, PP4
25	c.334 G>C	Hu et al. (2022)	1	Exon 6	MS	VUS	PP3, PP4
26	c.346 C>T	Yang et al. (2008)	1	Exon 7	MS	LP	PS3, PP3, PP4
27	c.364 G>C	Hu (2019)	1	Exon 7	MS	VUS	PP3, PP4
28	c.368 T>A	Hu et al. (2022)	1	Exon 7	MS	VUS	PM2, PP3, PP4
29	c.405_406del	Yang et al. (2020b)	1	Exon 7	FS	P	PVS1, PM2, PP4
30	c.410 T>C	Lam et al. (2011)	1	Exon 7	MS	VUS	PM2, PP3, PP4
31	c.423-2A>G	Hu (2019)	1	IVS 7	SD	VUS	PVS1, PP4
32	c.422 + 1G>A	Haiqing (2022)	1	IVS 7	SD	P	PVS1, PM2, PP4
33	c.445 C>T	Li et al., 2015a; Hu (2019)	4	Exon 8	NS	P	PVS1, PS3, PP4
34	c.446 G>C	Yang et al. (2008)	1	Exon 8	MS	LP	PS3, PM5, PP3, PP4
35	c.469 A>T	Hu (2019)	1	Exon 8	NS	P	PVS1, PM2, PP4
36	c.499 C>T	Haiqing (2022)	1	Exon 9	MS	LP	PS3, PM5, PP3, PP4
37	c.503 G>T	Gao et al. (2021)	1	Exon 9	MS	VUS	PP3, PP4
38	c.530 T>G	Hu et al. (2022)	1	Exon 9	MS	VUS	PP3, PP4
39	c.541 C>T	Huang et al. (2021)	1	Exon 9	NS	P	PVS1, PS3, PP4
40	c.579_583del	Haiqing (2022)	1	Exon 9	FS	P	PVS1, PM2, PP4
41	c.582_586del	Hu (2019)	1	Exon 9	FS	P	PVS1, PM6, PP4
42	c.583 C>T	Hu et al. (2022)	1	Exon 9	MS	LP	PS3, PP3, PP4
43	c.594 G>A	Yang et al. (2020a)	1	Exon 9	NS	P	PVS1, PM2, PP4
44	c.597dupC	Hu (2019)	1	Exon 9	FS	P	PVS1, PM6, PP4
45	c.517 C>T	Yang et al., 2008; Xie (2012), Chen et al. (2015), You et al. (2015), Yang et al., 2016; Hu (2019), Sun et al. (2020), Hu et al. (2022)	16	Exon 9	MS	P	PS3, PM5, PM1, PP3, PP4
46	c.518 G>A	Yuan et al. (2015), Li et al. (2017), Wang et al., 2019b; Hu (2019)	4	Exon 9	MS	P	PS3, PM5, PM1, PP3, PP4
47	c.518 G>C	Li (2022)	1	Exon 9	MS	P	PS3, PM5, PM2, PM1, PP3, PP4
48	c.648_651+1del	Zhou et al. (2022)	1	Exon 10	SD	P	PVS1, PM2, PP4
49	c.651 G>C	Yang et al. (2008)	1	Exon 10	MS	LP	PS3, PP3, PP4
50	c.651 + 2 A>G	Li et al. (2016)	1	IVS 10	SD	P	PVS1, PM2, PP4
51	c.652-1 G>A	Yang et al. (2008)	1	IVS 10	SD	VUS	PVS1, PP4
52	c.652 G>A	Yang et al. (2008)	1	Exon 11	MS	LP	PS3, PM2, PP3, PP4
53	c.655 G>C	Li et al. (2015b)	1	Exon 11	MS	VUS	PM2, PP3, PP4
54	c.653 G>A	Hu, 2019; Hu et al. (2022)	2	Exon 11	MS	LP	PM2, PM6, PP3, PP4
55	c.655_656insG	Yang et al. (2008)	1	Exon 11	FS	P	PVS1, PS3, PP4
56	c.657_658del	Wang et al. (2019a)	1	Exon 11	FS	VUS	PVS1, PP4
57	c.662 G > A	Yang et al., 2008; Hu (2019)	2	Exon 11	MS	LP	PS3, PM2, PP3, PP4
58	c.673 C>T	Hu, 2019; Zhang et al. (2020b), Hu et al. (2022)	11	Exon 11	NS	VUS	PVS1, PP4
59	c.713 T>C	Zhang and Gao (2019)	1	Exon 11	MS	VUS	PP3, PP4
60	c.673_674del	Hu (2019)	1	Exon 11	FS	P	PVS1, PM2, PP4
61	c.726_727del	Hu (2019)	1	Exon 11	FS	P	PVS1, PM6, PP4
62	c.728_729del	Hu et al. (2022)	1	Exon 11	FS	VUS	PVS1, PP4
63	c.730_731del	Hu, 2019; Li (2022)	2	Exon 11	FS	P	PVS1, PS3, PP4
64	c.734_741del	Hu (2019)	1	Exon 11	FS	P	PVS1, PM6, PP4
65	c.739 T>C	Hu et al. (2022)	1	Exon 11	MS	LP	PS3, PP3, PP4
66	c.760del	Lam et al. (2001)	1	Exon 11	FS	P	PVS1, PM2, PP4
67	c.771 G>A	Hu, 2019; Li et al. (2022)	2	Exon 11	SD	VUS	PP4
68	c.741_748dupCATCGCTG	Zhou (2014)	1	Exon 11	FS	VUS	PVS1, PP4
69	c.760_771+2delinsGCTGCATCGCTGAA	Hu, 2019; Zhang et al. (2021)	2	Exon 11	SD	P	PVS1, PM6, PP4
70	c.771 + 1 G>C	Hu et al. (2022)	1	IVS 11	SD	VUS	PVS1, PP4
71	c.772-1 G>C	Yang et al. (2008)	1	IVS 11	SD	P	PVS1, PS3, PP4
72	c.772-17 A>G	Hu (2019)	1	IVS 11	SD	VUS	PP4
73	c.772-2 A>G	Kong et al. (2013)	1	IVS 11	SD	VUS	PVS1, PP4
74	c.806 C>G	Li et al. (2015a), Hu et al., 2018b; Hu (2019), Teng et al. (2020)	5	Exon 12	MS	LP	PS2, PP3, PP4
75	c.809del	Zheng et al. (2018)	1	Exon 12	FS	P	PVS1, PM2, PP4
76	c.847_848del	Hu (2019)	1	Exon 13	FS	VUS	PVS1, PP4
77	c.875_876del	Yang et al. (2017)	1	Exon 13	FS	P	PVS1, PM2, PP4
78	c.882del	Hu et al. (2022)	1	Exon 13	FS	P	PVS1, PM6, PP4
79	c.902_909del	Yang et al. (2008)	1	Exon 13	FS	P	PVS1, PS3, PP4
80	c.848G>A	Yang et al. (2016)	2	Exon 13	NS	VUS	PVS1, PP4
81	c.912 + 1 G>C	Hu (2019)	1	IVS 13	SD	VUS	PVS1, PP4
82	c.913-2 A>G	Li et al., 2015a; Hu (2019), Hu et al. (2022)	3	IVS 13	SD	VUS	PVS1, PP4
83	c.913-9_914del	Hu et al. (2022)	1	Exon 14	SD	P	PVS1, PM2, PP4
84	c.936_937insTGAC	Yang et al. (2008)	1	Exon 14	FS	P	PVS1, PS3, PP4
85	c.962G>A	Hu et al. (2022)	1	Exon 14	MS	VUS	PP3, PP4
86	c.963_964insT	Yang et al. (2008)	1	Exon 14	FS	P	PVS1, PS3, PP4
87	c.973C>T	Yang et al. (2008), Li et al., 2015b; Hu (2019)	6	Exon 14	NS	P	PVS1, PS3, PP4
88	c.988G>C	Yang et al. (2015), Yang et al. (2016)	3	Exon 14	MS	VUS	PM2, PP3, PP4
89	c.992C>T	Zhang and Gao (2019), Hu et al. (2022)	1	Exon 14	MS	VUS	PP3, PP4
90	c.994C>T	Hu et al. (2022)	1	Exon 14	NS	VUS	PVS1, PP4
91	c.1005dupC	Yang et al. (2022)	1	Exon 14	FS	P	PVS1, PM2, PP4
92	c.1008_1019del	Yang et al. (2008)	1	Exon 14	FS	P	PVS1, PS3, PM2, PP4
93	c.1045_1046del	Hu (2019)	1	Exon 14	FS	P	PVS1, PS3, PM6, PM2, PP4
94	c.1071del	Li et al. (2015a)	1	Exon 14	FS	P	PVS1, PS2, PP4
95	c.1078_1132del	Li (2022)	1	Exon 14	FS	P	PVS1, PS3, PM2, PP4
96	c.1070C>A	Xie (2012)	1	Exon 14	MS	VUS	PM2, PP3, PP4
97	p.(Asp359Asn)	Li et al. (2015a)	1	Exon 14	MS	VUS	PP3, PP4

MS = missense; SD = splice defect; FS = frameshift; NS = nonsense; VUS = uncertain significance; LP = likely pathogenic; P=Pathogenic; PVS1 = Nonsense/frameshift/canonical ±1 or 2 splice sites; PS2 = de novo variant; PS3 = *in vitro* or *in vivo* functional studies supportive of a damaging effect on the gene or gene product; PM1 = Located in a mutational hot spot and/or critical and well-established functional domain; PM2 = Variant was neither found in ExAC, nor 1000G and ESP; PM5 = novel missense change at an amino acid residue where a different missense change determined to be pathogenic has been seen before; PM6 = assumed *de novo*, but without confirmation of paternity and maternity.

### 3.3 Clinical characteristic of Chinese people with AIP

Clinical data were clearly reported for 77 out of 160 patients. Among the 77 AIP patients, there were 65 females (female/male ratio: 6.7:1), with an average age of 28.8 ± 9.9 years (range: 1–59 years), and the majority (83.1%) were aged between 18-39 years old (Table 2). The most common symptom was abdominal pain (73/77, 94.8%), often accompanied with nausea, anorexia, vomiting, constipation, and ileus. 58.4% (45/77) of AIP patients suffered from central nervous system symptoms, such as consciousness disturbances, seizures, respiratory paralysis, dizziness, headache, and dysphagia. One patient presented with dizziness and was diagnosed with “acute cerebral infarction,” while another patient presented with consciousness disturbances being diagnosed with “intracranial hemorrhage.” 32.2% (24/77) of patients experienced peripheral nervous system symptoms, such as weakness, numbness or stiffness in limbs, somatic pain, and limb paralysis. 13.0% (10/77) of patients experienced psychiatric symptoms, such as anxiety, depression, irritability, hallucination, and delirium. 48.1% (37/77) of patients suffered from autonomic nervous system symptoms, such as tachycardia, hypertension, urinary retention, and hypotension. Hyponatremia was the most common laboratory abnormality (42/77). Other abnormalities observed were liver dysfunction (18/77), kidney dysfunction (8/77), anemia (13/77), hypokalemia (3/77), sex hormonal imbalance (2/77), cortisol rhythm disturbance (1/77). Urinary PBG testing was performed on 38 patients, all results were positive (Table 2). Treatment strategy and outcome were reported in 42 patients (Table 2). 31 patients received the administration of high glucose, and 30 of them were improved. One severe patient with respiratory paralysis died. 6 patients were treated with high glucose combined with hemin therapy, 5 eventually improved and one died due to multi-organ failure. One case was complicated by depression, and after effective antidepressant treatment, her AIP symptoms were also controlled. After receiving prophylactic hemin infusion treatment, two patients exhibited a reduction in the frequency of acute attacks. In addition, three patients who experienced menstrual-associated acute attacks were administered gonadotropin-releasing hormone analogue (GnRH-a) for menstrual suppression to mitigate these episodes. Symptomatic treatments including fluid restriction, sedation, analgesia, hepatoprotection, and antiepileptic therapy were provided based on individual patient conditions.

**TABLE 2:** Clinical data of 77 Chinese patients with AIP.

Cite	No. cases	Age	Sex	Mutation	Triggers	Digestive system	Neuropsychiatric system			
							CNS	ANS	PNS	Psychiatric symptom
Lam et al. (2001)	1	40	F	c.760del	-	Abdomen pain	Convulsions	-		
Yang et al. (2008)	2–25	31.9 ± 7.7	F (21) M (3)	-	-	Abdomen pain (24), nausea, vomiting, constipation	consciousness disturbances, seizures (9)	Orthostatic hypotension tachycardia, hypertension (4)	-	-
Lam et al. (2011)	26	39	F	c.410 T>C	-	-	Dizziness	Hypertension	Fatigue	hallucination
Xie (2012)	27	28	F	c.517 C>T	Menstruation	Abdomen pain, nausea, abdominal distension	consciousness disturbances, seizures	-	-	-
Kong et al. (2013)	28	32	F	c.772–2A>G	-	Abdomen pain, vomiting, abdominal distension	Consciousness disturbances, seizures	Tachycardia hypertension	numbness in limbs	-
Zhou (2014)	29	36	F	c.741_748dupCATCGCTG	Menstruation, drinking	Abdomen pain, nausea, abdominal distension	Consciousness disturbances, seizures	-	numbness in limbs	-
Li et al. (2015b)	30	26	F	c.973C>T	Tired	Abdomen pain, nausea, vomiting	seizures	Hypertension	-	-
	31	32	F	c.655G>C	Menstruation	Abdomen pain	-	Hypertension	-	-
Yang et al. (2015)	32	21	F	c.988G>C	Menstruation	Abdomen pain, vomiting, ileus	Consciousness disturbances, seizures	-	-	-
Chen et al. (2015)	33	39	M	c.517C>T	smoking	Abdomen pain, constipation	Seizures	Tachycardia, hypertension	fatigue	-
Jiao et al. (2015)	34	23	F	c.88–2A>G	Menstruation	Abdomen pain	-	-	muscle stiffness	delirium
Yuan et al. (2015)	35	45	M	c.518G>A	Drinking	Abdomen pain, nausea, vomiting, and constipation	-	-	lower limbs muscle pain, weakness	irritability, hallucinations, delirium
You et al. (2015)	36	20	F	c.517C>T	Tired Menstruation	Abdomen pain, nausea, vomiting, abdominal distension, constipation	Seizures	Tachycardia	fatigue	-
Yang et al. (2016)	37	21	F	c.988G>C	-	Abdomen pain constipation	Consciousness disturbances, seizures	Tachycardia	-	-
	38	31	F	c.988G>C	-	Abdomen pain constipation	-	-	-	-
	39	45	M	c.517C>T	-	Abdomen pain	-	-	-	-
	40	26	F	c.517C>T	-	Abdomen pain constipation	-	-	-	-
	41	28	F	c.517C>T	-	Abdomen pain	Consciousness disturbances, seizures	-	-	-
	42	22	F	c.848G>A	-	Abdomen pain constipation	Respiratory paralysis	-	-	-
	43	23	F	c.848G>A	-	Abdomen pain constipation	Consciousness disturbances, seizures	Tachycardia	-	-
	44	34	F	c.517C>T	-	Abdomen pain	Consciousness disturbances, seizures	Tachycardia	-	-
	45	24	F	c.806C>G	-	Abdomen pain, vomiting, constipation	-			
	46	22	F	c.1071del	-	Abdomen pain, vomiting, constipation	-			
Li et al. (2016)	47	24	F	c.806C>G	-	Abdomen pain, vomiting, constipation	-	-	-	-
Yang et al. (2017)	48	28	F	c.875_876del	Fast	-	Seizures	-	Fatigue, pain in limbs	
Lei et al. (2017)	49	27	F	c.100C>T	Tired	Abdomen pain, vomiting, ileus	Consciousness disturbances, seizures, dysphagia, headache, respiratory paralysis	Tachycardia	numbness of limbs	-
Li et al. (2017)	50	25	F	c.518G>A	-	Abdomen pain constipation	Dysphagia, respiratory paralysis	-	Fatigue, numbness of limbs	Depression
Hu et al. (2018b)	51	23	F	c.806C>G	Menstruation	Abdomen pain, nausea, vomiting, abdominal distension, constipation	-	Tachycardia	Fatigue, numbness of limbs	-
Zheng et al. (2018)	52	28	M	c.809del	-	Abdomen pain, nausea, constipation	Consciousness disturbances, seizures	Tachycardia, hypertension	-	-
Zhang and Gao (2019)	53	22	F	c.992C>T	Menstruation	Abdomen pain, diarrhea, constipation	-	-	-	-
	54	59	M	c.713T>C	-	Abdomen pain, nausea, and anorexia	-	Hypertension	-	-
Wang et al. (2019a)	55	24	F	c. 657_658del	Pregnancy	Abdomen pain, vomiting, diarrhea	Consciousness disturbances, intracranial hemorrhage	-	-	-
Wang et al. (2019b)	56	24	F	c.518G>A	-	Abdomen pain, nausea, vomiting, anorexia, ileus	Consciousness disturbances, seizures	Tachycardia, hypertension	Fatigue, Tendon reflex not elicited	-
	57	24	F	c.518G>A	-	Abdomen pain, abdominal distension	-	Tachycardia	-	-
Yang et al. (2020b)	58	36	F	c.405_406del	Pregnancy	Abdomen pain	Seizures, blurred vision	-	Limb paralysis	impaired speech and memory
Sun et al. (2020)	59	20	F	c.517C>T	Menstruation	Abdomen pain, vomiting, diarrhea, abdominal distension	Consciousness disturbances, seizures	Tachycardia, hypertension	-	Anxiety, depression, hallucination
Yang et al. (2020a)	60	20	F	c.594G>A	-	Abdomen pain, nausea, vomiting, ileus	Consciousness disturbances, seizures	-	-	
Zhang et al. (2020b)	61	32	F	c.673C>T	Sex hormone change	Abdomen pain, nausea, vomiting, constipation	Respiratory paralysis	-	-	Depression
Teng et al. (2020)	62	30	F	c.806C>G	Pregnancy	Abdomen pain, nausea, vomiting, constipation	Seizures, blurred vision, headache	Hypertension	-	-
Huang et al. (2021)	63	37	F	c.541C>T	-	Abdomen pain, abdominal distension, ileus	Consciousness disturbances, respiratory paralysis	Tachycardia Hypertension	Fatigue, unspecific peripheral nerve damage	-
Fu et al. (2021)	64	37	F	c.181G>C	-	Abdomen pain, nausea, vomiting	Seizures, headache	Tachycardia Hypertension	-	Depression
Gao et al. (2021)	65	54	M	c.503C>T	-	Abdomen pain, nausea, anorexia	Dizziness, cerebral infarction	Hypertension	Fatigue, peripheral nerve damage, numbness of limbs	-
Zhou et al. (2022)	66	22	F	c.648_651+1del	Menstruation, Pregnancy	Abdomen pain	Seizures	Tachycardia Hypertension	Weakness in lower limbs	-
Yang et al. (2022)	67	23	F	c.1005dupC	-	Abdomen pain, constipation	Seizures	Tachycardia	Quadriparesis	-
Zho et al. (2022)	68	4	F	c.579_583del	-	-	Seizures	-	-	-
Li et al. (2022)	69	20	F	c.331G>A	Menstruation, tired	Abdomen pain, nausea, vomiting	Respiratory paralysis	Tachycardia	-	Depression
Li (2022)	70	20	F	c.1078_1132del	Tired, irregular diet	Abdominal pain, nausea, vomiting, abdominal distension, constipation	Consciousness disturbances, seizures	Tachycardia Hypertension	Fatigue, stiffness on both front thighs and lower back	-
Li (2022)	71	23	F	c.160 + 5G>C	Menstruation	Abdominal pain, nausea, vomiting, abdominal distension, constipation	-	Tachycardia Hypertension	Fatigue	-
Li (2022)	72	20	F	c.730_731del	Menstruation, Tired, irregular diet	Abdominal pain, nausea, vomiting, constipation, ileus	seizures	Tachycardia Hypertension	Fatigue, stiff hands, numbness in limbs	-
Li (2022)	73	19	F	c.518G>C	Menstruation, irregular diet	Abdominal pain, nausea, vomiting, constipation	Consciousness disturbances, seizures	Tachycardia Hypertension	Fatigue, numbness and pain in hands	-
Liang and Li (2023)	74	18	F	c.76C>T	-	Abdomen pain, anorexia, constipation	-	Tachycardia Hypertension	Fatigue	Depression
Haiqing (2022)	75	1	F	c.579-583del	-	-	seizures	Tachycardia	-	-
	76	1	M	c.499C>T	-	-	Consciousness disturbances, seizures	Tachycardia	-	-
	77	5	F	c.422 + 1G>A	-	Abdomen pain, vomiting	-	Tachycardia	-	-

Hyponatremia	Endocrine system	Other	Urine PBG	Recurrent	Therapy	Outcome
-	-	Anemia	+	Yes	-	-
+^a^	-	Urinary retention (4)	+	-	-	-
+	Diabetes	respiratory distress, multi-organ failure	+	-	Glucose, hemin	Death
+	-	-	+	Yes	Glucose	Recovered
+	-	CRF, Anemia, renal dysfunction	+	Yes	-	-
-	-	Muscle and joint pain	-	Yes	Glucose	Recovered
+	-	-	+	Yes	-	-
+	-	-	+	Yes	-	-
+	-	Anemia	+	Yes	Glucose	Recovered
+	-	Liver dysfunction, rhabdomyolysis	+	Yes	-	-
+	-	Liver dysfunction	+	Yes	-	-
+	-	Urinary retention	+	Yes	-	-
+	-	liver and kidney dysfunction	+	Yes	Glucose	Recovered
+	-	-	+	Yes	Glucose	Recovered
+	-	-	+	Yes	Glucose	Recovered
+	-	Urine retention	+	Yes	Glucose	Recovered
+	-	-	+	Yes	Glucose	Recovered
+	-	-	+	Yes	Glucose	Recovered
+	-	-	+	Yes	Glucose	Death
+	-	-	+	Yes	Glucose	Recovered
+	-	Sun sensitivity	+	Yes	Glucose	Recovered
+		Anemia, Liver dysfunction	-	YES	-	-
+		Anemia, Liver dysfunction	-	YES	-	-
+	-	Anemia, Liver dysfunction	-	YES	-	-
+	-	Respiratory failure	+	Yes	Glucose	Recovered
+	-	Liver dysfunction	-	Yes	Glucose	Recovered
-	-	Anemia, Liver dysfunction	+	Yes	Glucose	Recovered
+	Hypokalemia	-	+	Yes	Glucose, GnRH-a	Recovered
+	-	Anemia, kidney dysfunction	+	Yes	Glucose	Recovered
-	-	-	+	Yes	Preventive infusion of hemin	Recovered
-	-	Backache	+	Yes	Glucose, hemin, Preventive infusion of hemin	Recovered
-	-	-	-	-	Glucose	Recovered
+	Hyperandrogenemia, cortisol rhythm disturbance, Hypokalemia	Liver dysfunction, Hyperlipidemia	-	-	Glucose	Death
-	Elevated FT4	Anemia, liver and kidney dysfunction	-	Yes	Glucose	Recovered
-	hypoglycemia	Liver dysfunction	+	Yes	Glucose	Recovered
+	-	Fever, Anemia	-	Yes	Glucose	Recovered
+	-	-	+	-	Glucose	Recovered
-	Sexual hormone disorder, Turner syndrome	primary amenorrhea	-	Yes	Antidepressant	Recovered
-	Hashimoto’s thyroiditis	Chest and back pain, Sjogren’s syndrome	+	Yes	Glucose hemin	Recovered
-	-	Kidney dysfunction	-	Yes	Glucose	Recovered
+	-	Kidney dysfunction, rash	+	Yes	Glucose	Recovered
-	-	Anemia, kidney dysfunction	-	Yes	Glucose	Recovered
+	-	Liver dysfunction	+	Yes	Glucose	Recovered
+	Low T3	Femoral pain, anemia	+	Yes	Glucose	Recovered
-	-	-	-	Yes	-	-
-	-	Fever, kidney dysfunction, DIC, AHF	+	Yes	Glucose, hemin	Recovered
+	-	Liver dysfunction	+	Yes	Glucose	Recovered
+	-	Liver dysfunction	+	-	Glucose	Recovered
+	-	Liver dysfunction	+	Yes	Glucose	Recovered
+	-	Liver dysfunction	+	Yes	Glucose	Recovered
+	Hypokalemia	Liver dysfunction	-	Yes	Glucose, hemin	Recovered
-	-	-	-	Yes	Symptomatic treatment	Recovered
-	-	-	-	Yes	Symptomatic treatment	Recovered
-+	Hypokalemia	Fever, Liver dysfunction, anemia	+	Yes	Symptomatic treatment	Recovered

CNS, central nervous system; PNS, peripheral nervous system; ANS, autonomic nervous system; PBG, porphobilinogen; ^a^ = 5 (5/24) patients have hyponatremia; - = no information available for this item; DIC = disseminate intravascular coagulation; AHF , acute heart failure; CRF = chronic renal failure.

### 3.4 Genotype-phenotype association of Chinese people with AIP

A total of 53 Chinese AIP patients with 36 variants were included in the genotype-phenotype association analysis (reported cases 2–25 were excluded because the variants did not match with the patient’s phenotype). As shown in Tables 1, 3, patients with nonsense variants (c.100C>T, c.541C>T, c.594G>A, c.673C>T, c.848G>A, and c.973C>T) had only moderate and severe phenotypes. Patients with frameshift variant (c.405_406del, c.579_583del,c.648_651+1del,c.730_731del,c.760del,c.741_748dupCATCGCTG, c.809del, and c.1005dupC) mainly experienced moderate phenotype, while c. 657_658del and c.875_876del were found in severely affected patients and 1071del was found in mildly affected patient. And patients with missense variant and splice defect were associated with mild, moderate and severe phenotype, while affecting enzyme activity center (c.76C>T, c.517C>T, c.518G>A, and c.518G>C) mainly experienced moderate and severe phenotype.

**TABLE 3:** Genotype-phenotype association of Chinese AIP patient.

Variants	Type	No. of patients with phenotype		
		Mild	Moderate	Severe
c.405_406del	FS		1	
c.579_583del	FS		2	
c.648_651+1del	FS		1	
c. 657_658del	FS			1
c.730_731del	FS		1	
c.760del	FS		1	
c.741_748dupCATCGCTG	FS		1	
c.809del	FS		1	
c.875_876del	FS			1
c.1005dupC	FS		1	
c.1071del	FS	1		
c.1078_1132del	FS		1	
c.76C>T	MS		1	
c.181G>C	MS		1	
c.331G>A	MS			1
c.410 T>C	MS			1
c.499C>T	MS		1	
c.503C>T	MS			1
c.517 C>T	MS	1	6	1
c.518G>A	MS		4	1
c.518G>C	MS			1
c.655G>C	MS	1		
c.713T>C	MS	1		
c.806C>G	MS	2	2	
c.988G>C	MS	1	1	
c.992C>T	MS	1		
c.100C>T	NS			1
c.541C>T	NS			1
c.594G>A	NS		1	
c.673C>T	NS			1
c.848G>A	NS		1	1
c.973C>T	NS		1	
c.88-2A>G	SD		1	
c.160+5G>C	SD	1		
c.422+1G>A	SD	1		
c.772-2A>G	SD			1

MS = missense; SD = splice defect; FS = frameshift; NS = nonsense.

## 4 Discussion

This is the first study to provide a comprehensive description of the clinical and genetic features of Chinese patients with AIP. The findings of this study may have significant implications for the management of this disease. Identifying the most common clinical features can help clinicians to recognize AIP patients across different departments. Since the symptoms of AIP are not specific, diagnosis is usually delayed (Anderson et al., 2005), and molecular analysis of the *HMBS* gene has become the most useful diagnostic test for identifying asymptomatic AIP family members and those in the intermission of attacks. Improved understanding of the molecular heterogeneity of the *HMBS* gene can help clinicians provide patients with clinical counseling and health education to prevent life-threatening acute attacks. Additionally, genotype-phenotype association analysis may predict severe clinical phenotypes in future patients.

The clinical manifestations of AIP are diverse and are characterized by life-threatening acute intermittent attacks, caused by porphyrin accumulation in the visceral, central, peripheral, and autonomic nervous systems (Stölzel et al., 2021). Abdominal pain is the most common clinical manifestation during AIP attacks (Ramanujam and Anderson, 2015), and it is also the first symptom to appear (Kothadia et al., 2023). A large population survey in the United States in 2020 showed that approximately 1/4 of patients with a history of abdominal pain had symptoms similar to acute hepatic porphyria (Lakhoo et al., 2021). These gastrointestinal symptoms can often cause decreased appetite and impaired energy intake and absorption, resulting in a negative energy balance in patients, which in turn further exacerbates the onset of AIP. 94.8% (73/77) of the AIP patients included in this study experienced abdominal pain, often accompanied by nausea, vomiting, abdominal distension, and constipation. A prospective, multinational, natural History study showed abdominal pain is the most common symptom during acute attacks (92%) (Gouya et al., 2020), which is consistent with our results. However, the incidence of abdominal pain in the Chinese patients is significantly higher than that in Brazilian patients (77/172,52%) (Souza et al., 2023).

The second major clinical manifestation was neuropsychiatric symptoms, such as conscious disorder, convulsions, weakness, stiffness and numbness of the extremities, anxiety, depression, irritability, hallucinations, and delirium, and the convulsions in some patients may be related to hyponatremia. AIP should be remembered as an important differential diagnosis for neuromuscular disorders (de Souza et al., 2021). In our study, hyponatremia is the most common biochemical abnormality, 55.5% of patients had hyponatremia which may be associated with syndrome of inappropriate antidiuretic hormone secretion (SIADH) (Li et al., 2015; Yang et al., 2016). It has been shown that large accumulations of porphyrin precursors cause damage to the hypothalamus, resulting in increased secretion of vasopressin and retention of large amounts of body fluid, causing dilutional hyponatremia (Aksoy Ö et al., 2020). Therefore, the key to correcting hyponatremia in AIP patients is to limit their fluid intake.

Disorders of the endocrine metabolic system may arise due to abnormal porphyrin metabolism. Our study findings suggest that some patients with AIP might exhibit endocrine disorders, including hyperprolactinemia, hyperandrogenism, disruption of cortisol rhythms, thyroid dysfunction, and abnormal glucose metabolism. Limited research has been conducted on the impact of porphyrin metabolism on the endocrine system; however, it is hypothesized that these alterations could be associated with damage to the hypothalamus, pituitary gland, and other endocrine glands caused by porphyrin accumulation. The precise mechanism underlying these changes is still being investigated.

Glucose loading therapy is effective for most acute attacks. In China, the majority of patients recovered with intravenous glucose infusions. However, glucose can only control acute attacks, and there is a need to develop new specific medicine to treat severely patients and prevent acute attacks of AIP. Hemin is the first line therapy for AIP, and at present, Givosiran also has been emerged as first line therapy for AHP in the last years, however, in China, they are much expensive and difficult to obtain, therefore, it has not yet been put into clinical treatment for AIP patients, only a very small number of Chinese AIP patients receive intravenous hemin infusions to control and prevent acute attacks of AIP. In terms of treatment, avoiding triggers is the key to controlling acute attacks of AIP. Some patients in this study experienced acute attacks related to menstruation and depression, which were reduced with GnRH-a and antidepressant treatment. The majority of patients are able to recover after treatment, but a few severe patients may die due to prolonged delay or worsening of the disease. Therefore, early diagnosis and effective treatment are crucial for patient prognosis. Glucose-loading therapy has demonstrated efficacy in managing the majority of acute attacks. In China, intravenous glucose infusions have led to successful recovery in most patients. However, glucose alone can only control acute attacks, highlighting the urgent need for the development or introduction of new specific medications that can effectively treat severe cases and prevent acute attacks of AIP. Currently, hemin is considered the first-line therapy for AIP, while Givosiran has emerged as the first-line therapy for AHP in recent years. Nevertheless, these medications are often prohibitively expensive and difficult to access in China, which hinders their clinical use among AIP patients. Consequently, only a small fraction of Chinese AIP patients receive intravenous hemin infusions to manage and prevent acute attacks. To effectively manage such attacks, it is crucial to identify triggers and implement appropriate interventions. Notably, some patients in this study experienced acute attacks associated with menstruation and depression; however, these symptoms were alleviated through GnRH-a administration and antidepressant treatment respectively. While most patients achieve recovery after treatment initiation, a few severe cases may succumb due to delayed diagnosis or disease progression. Therefore, early diagnosis coupled with effective treatment strategies play a pivotal role in determining patient prognosis. According to the Human Gene Mutation Database (HGMD), more than 500 variants have been reported, the majority of which were missense mutations (31.9%), followed by small deletions, insertions and duplications (Bustad et al., 2021). A number of foreign studies also showed that missense mutations accounted for the largest proportion, which was consistent with our findings. In this study, 97 variants were detected in 160 unrelated families, including 35 missense, 29 frameshift, 24 splicing and 9 nonsense variants. We investigated the pathogenicity of 97 variants including in this study, among them, 45 variants are pathogenic, 14 variants are likely pathogenic and 38 variants are uncertain significance (Table 1). In China, most variants were reported in case reports, and there is insufficient function study on *HMBS* variants. Only 1/3 of the variants have undergone preliminary function study (Table 1). Further research is needed to elucidate the pathogenicity of these variants.

The distribution of variants in the *HMBS* gene exhibits some degree of variation among different countries. The number of variants in exons 9 and 11 exceeded others, mainly due to their size (Kauppinen and von und zu Fraunberg, 2002; Kauppinen, 2004). A study of 121 unrelated French Caucasian AIP families identified 78 different variants, 60% of which were in exons 9, 11, and 13 (Puy et al., 1997). In China, the variants in exons 11 and 14 were more widely distributed, demonstrating the heterogeneity (Figure 2). Most of the variants were family specific, that was, the occurrence of the same variant in several families was very low. In our study, c.517C>T occurred in 16 unrelated families, and 22 other variants also occurred in more than one family, and most of them are missense mutations (Figure 2). c.517C>T was a known pathogenic variant in Nova Scotia, Canada, with a high frequency due to the founder effect (Greene-Davis et al., 1997). In 2009, Sharon D identified 123 different variants on 283 patients in the UK, most variants were present in fewer than 3 families, but c.517C>T was present in 35 families (12%) (Whatley et al., 2009). It has also been reported that a variant was often shared by several families because of the founder effect, such as p. (Trp198*) from Sweden, p. (Gly111Arg) from Argentina, p. (Trp283*) from Switzerland, c. 669_698del from Spain (Guillén-Navarro et al., 2004), p.Arg116Trp from the Netherlands (de Rooij et al., 2009).

The correlation between genotype and phenotype should be cautiously interpreted, considering the clinical phenotype observed in *HMBS* gene variants (Table 2), which highlights the impact of genotype on phenotype. Variations in clinical presentation among patients carrying the same variant may suggest the involvement of modifier genes or environmental factors. Previous studies on glutathione synthetase deficiency have demonstrated that mutations leading to frameshifts, premature stop codons, or aberrant splicing are associated with moderate to severe clinical phenotypes (Njålsson et al., 2005). Consistent with these findings, our study also reveals that variants causing premature stop codons, frameshifts, or disruption of enzyme activity center are more likely to result in severe clinical manifestations such as respiratory paralysis, intracranial hemorrhage, disseminated intravascular coagulation (DIC), acute hepatic failure (AHF), or respiratory failure.

Among all the AIP patients we collected, there were obvious regional differences. They were mainly reported from Hebei, Taiwan and Beijing, and almost all the reports came from third-class hospitals. The regional medical level was a significant factor. Many patients chose to go to first-class hospitals because of unclear diagnosis and recurrent attacks. This suggests that we still lack awareness of this disease, especially in primary hospitals. In addition, our research was based only on *HMBS* variants from literature reports, so it was highly influenced by reporting bias. Further epidemiological data may provide more accurate information. Although there were some disadvantages in our study, this was the first investigation of *HMBS* gene variant in China, which fully revealed the characteristics of *HMBS* variant.

In the process of literature retrieval, we found that many patients with a clinical diagnosis of AIP did not receive related genetic testing; all the patients had clinically proven elevated urinary porphyrin precursors. Most patients recovered with carbohydrate intake. Therefore, variant screening was not necessary for patients with clinical onset and was not recommended in the normal population. However, in addition to AIP, other hepatic porphyria such as hereditary coproporphyrin (HCP) and variegate porphyria (VP) also had elevated urinary porphyrin precursors, so it had limitations in identifying of acute porphyria (Stölzel et al., 2019). More importantly, it might be the only way for early diagnosis of other asymptomatic patients in AIP family. Thus, large-scale mutation screening was recommended among the AIP family members of a proband case. This might be especially true in populations where no founder effects has been identified in AIP patients (Kauppinen, 2004).

This study provides a preliminary analysis of the genetic and phenotypic characteristics of Chinese AIP patients. Our results increase clinicians’ understanding of AIP, which could provide clinical guidance for AIP patients and reduce misdiagnosis and mistaken treatment in China. However, whether these variants are pathogenic, further verification at the molecular level is required to provide a reliable basis for the clinical diagnosis, and might provide new ideas and methods for the treatment of acute attacks and long-term complications of AIP. At present, the research on rare diseases in China was still in the preliminary stage, so we should enhance the knowledge of this disease in both clinical and scientific research, and further develop a nationwide epidemiological survey of AIP, which will be a difficult task for us in the future.

There are certain limitations in this study. Firstly, due to the inherent nature of this systematic review, it is inevitable to encounter publication bias. Secondly, the analysis of clinical features did not include many Chinese AIP patients diagnosed solely by biochemistry, as a clear *HMBS* variant was lacking. Therefore, the data for analyzing clinical features may not be sufficiently comprehensive.

## 5 Conclusion

The analysis of the *HMBS* gene in Chinese AIP patients revealed that missense mutations were the most common variant, with c.517C>T being the predominant alteration. However, due to the strong heterogeneity of AIP, screening for a single variant is not recommended in suspected patients; instead, whole gene sequencing should be performed. Abdominal pain was identified as the most frequent presenting symptom, followed by central nervous system manifestations. Women aged 18–40 years with recurrent abdominal pain and/or neuropsychiatric symptoms associated with menstruation or dietary factors should be alerted to the possibility of AIP. Variants causing premature stop codons, frameshifts or enzyme activity center disorder may lead to more severe clinical phenotypes such as respiratory paralysis, intracranial hemorrhage, DIC, AHF, CRF or respiratory failure.

## Data Availability

The original contributions presented in the study are included in the article/Supplementary Material, further inquiries can be directed to the corresponding authors.
